# A Literature Review of Cost-Benefit Analyses for the Treatment of Alcohol Dependence

**DOI:** 10.3390/ijerph8083351

**Published:** 2011-08-16

**Authors:** Svetlana Popova, Satya Mohapatra, Jayadeep Patra, Amy Duhig, Jürgen Rehm

**Affiliations:** 1 Social and Epidemiological Research Department, Centre for Addiction and Mental Health, 33 Russell St., Toronto, Ontario M5S 2S1, Canada; E-Mails: satyacamh@gmail.com (S.M.); jaydeep.patra@gmail.com (J.P.); jtrehm@aol.com (J.R.); 2 Dalla Lana School of Public Health, University of Toronto, 155 College Street, Toronto, Ontario M5T 3M7, Canada; 3 Factor-Inwentash Faculty of Social Work, University of Toronto, 246 Bloor St. West, Toronto, Ontario M5S 1V4, Canada; 4 Eli Lilly and Company, Global Health Outcomes, Indianapolis, IN 46285, USA; E-Mail: amduhig@gmail.com; 5 Klinische Psychologie und Psychotherapie, Epidemiological Research Unit, Technische Universität, Hohe Street 53, Dresden D-01187, Germany

**Keywords:** alcohol dependence treatment, psychotherapy, pharmacotherapy, costs, benefits

## Abstract

The purpose of this study was to conduct a literature review of cost-benefit studies on pharmacotherapy and psychotherapy treatments of alcohol dependence (AD). A literature search was performed in multiple electronic bibliographic databases. The search identified seven psychotherapy studies from the USA and two pharmacotherapy studies from Europe. In the psychotherapy studies, major benefits are typically seen within the first six months of treatment. The benefit-cost ratio ranged from 1.89 to 39.0. Treatment with acamprosate was found to accrue a net benefit of 21,301 BEF (528 €) per patient over a 24-month period in Belgium and lifetime benefit for each patient in Spain was estimated to be Pta. 3,914,680 (23,528 €). To date, only a few studies exist that have examined the cost-benefit of psychotherapy or pharmacotherapy treatment of AD. Most of the available treatment options for AD appear to produce marked economic benefits.

## 1. Introduction

There are many types of treatment for alcohol dependence (AD), including psychosocial support groups such as Alcoholics Anonymous, inpatient and outpatient treatment, psychological interventions, pharmacological treatment, employee assistance programs (EAPs) and most typically, a combination of the aforementioned [1,2]. Most psychosocial interventions (e.g., cognitive behavioural therapy, motivational enhancement therapy) focus on helping patients decrease the frequency of alcohol use, and also address issues that have maintained their drinking behaviours such as familial, social, and work-related dynamics [3–5]. Psychosocial intervention formats are either one-on-one individual counselling or group counseling.

Pertaining to pharmacotherapies, the U.S. Food and Drug Administration (FDA) has approved four pharmacologic agents for the treatment of AD to date: disulfiram, oral naltrexone, injectable long-acting naltrexone, and acamprosate. The European Medicines Agency has not yet approved long-acting naltrexone.

Disulfiram, an aversive agent, has been used to treat AD for more than 50 years; however, the evidence for its effectiveness is weak. It has significant adverse effects and there is not sufficient evidence that it increases abstinence rates, decreases relapse rates, or reduces cravings [6]. Per the FDA, oral Naltrexone is indicated in the treatment of AD in combination with an appropriate plan of management for alcohol addiction. Over 20 clinical trials, as well as meta-analytic reviews support a modest effect of oral naltrexone, and support its effect on reducing heavy drinking, increasing abstinence rates and decreasing alcohol cravings in a number of study populations (e.g., [7–9]). Long-acting injectable naltrexone is indicated for the treatment of AD in conjunction with psychosocial support for patients who are abstinent at treatment initiation. Reductions in the number of drinking days and heavy drinking days have been reported; similar to oral Naltrexone, the effects are small [10].

In contrast to naltrexone, acamprosate is indicated for the maintenance of abstinence from alcohol in patients who are abstinent from consuming alcohol and who are simultaneously engaged in psychosocial support. Recent reviews of acamprosate in clinical trials suggest that the medication is primarily effective in extending continuous abstinence (e.g., [8,11]). Some other medications, although not approved by the U.S. FDA for the treatment of AD, have some support for their efficacy, such as anticonvulsants (e.g., gabapentin, topiramate [12], baclofen [13],), serotonergic agents (e.g., sertraline [14,15], ondansetron [12]), and glutamatergic agents (e.g., mementine; [6,16]).

To date, there are only a few studies that have examined the costs/benefits of psychotherapy or pharmacotherapy treatment of AD (see for example, [17]). A Cost-Benefit Analysis (CBA) incorporates multiple outcome measures to gain a more comprehensive picture of the total economic impact of the AD treatment. This analysis allows valuing of all outcomes of treatment in monetary terms so that the net economic benefits or a benefit-cost ratio, both of which enable the comparison of different treatment options, can be obtained. The purpose of the current study was to conduct a search of the current literature in order to determine the available evidence of cost-benefit analyses on pharmacotherapy and psychotherapy AD treatments.

## 2. Materials and Methods

### 2.1. Literature Search

A literature search for studies that have conducted a CBA on the social costs/benefits attributable to AD treatment was performed in multiple electronic bibliographic databases from January 1995 to January 2011, including: Ovid MEDLINE, PubMed, EMBASE, Web of Science, and PsychINFO. Google Scholar, the Cochrane Database of Systematic Reviews and economic databases, such as Centre for Review and Dissemination (CRD) (http://www.crd.york.ac.uk/crdweb/) were also searched. The search was conducted using multiple combinations of the following key words: alcohol, alcoholism, cost, dependence, treatment, pharmacotherapy, psychotherapy, psychosocial treatment, cost-benefit analysis, and economic evaluation. The search was not limited to English language publications or to any geographic area.

### 2.2. Data Extraction

Three investigators (S.M., J.P., and S.P.) independently extracted information from the identified studies. Training of coders to achieve sufficient (>0.80) interrater reliability (IRR) was conducted. IRR, a statistical measure for the degree of agreement among raters that gives a score of how much homogeneity, or consensus there is in the ratings given by different raters, was calculated by Fleiss’ kappa statistics using the attribute agreement analytic method. Discrepancies were reconciled by a fourth investigator (JR) independent of the first process. All analyses related to IRR were computed using Minitab ® statistical software [18]. Using a standardized spreadsheet (MS-Excel), each study was coded for the following variables: reference, country where the study was completed, sample size, type of intervention, types of cost, types of benefits, and benefit-cost ratio.

## 3. Results

**Figure 1.**
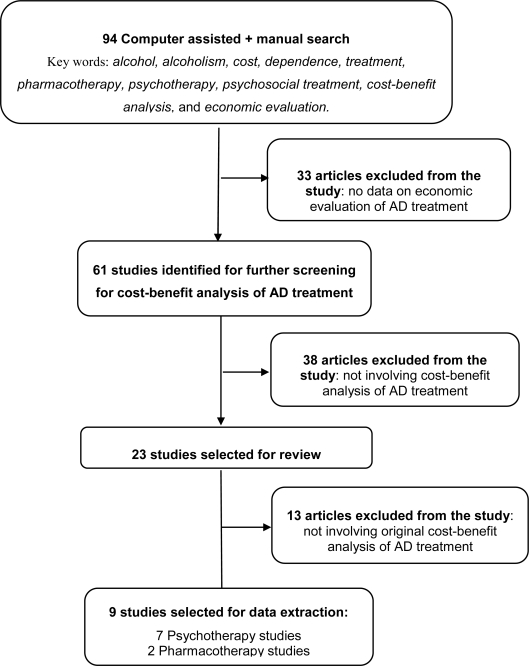
Flow diagram describing the search strategy for cost-benefit studies of alcohol dependence treatment.

In total, 94 articles were identified. After reviewing these articles, 61 articles were retained that included economic evaluations of alcohol treatment. Upon further screening for studies involving CBA of AD treatment, the data were extracted from nine articles providing original studies on the cost-benefit of AD treatment: seven psychotherapy studies from USA and two pharmacotherapy studies from Europe (one each from Belgium and Spain). A flow diagram describing the search strategy is presented in Figure 1.

There was a high IRR (j = 0.81, P < 0.0001) among the three reviewers across all variables coded. Table 1 provides the type of intervention and cost descriptions for the identified studies. The net benefits and/or benefit-cost ratios are presented in Table 2.

### 3.1. Psychotherapy Studies

In a study in Massachusetts, USA [20] involving 36 newly abstinent married male alcoholics, the patients were divided into three groups for outpatient treatment, namely: (1) Individual counselling; (2) Individual counselling plus behavioural marital therapy (BMT); and (3) Individual counselling plus interactional couples therapy (ICT) with a 24-month follow-up. It was observed that both individual counselling alone and in combination with BMT showed substantial and significant cost savings from reduced utilization of healthcare and legal systems that substantially and significantly exceeded the cost of delivering the treatment. Individual counselling alone had a higher benefit-cost ratio (20.77) than BMT plus individual counselling (8.64) due to the lower cost of delivering the treatment. Individual counselling plus ICT had a negative benefit-cost ratio of (−2.82) due to the high cost of treatment.

Another study by the above research group [20] involving 59 couples with a newly abstinent alcoholic husband, estimated the cost-benefit of BMT with or without relapse prevention (RP) sessions for alcoholics and their spouses. Both standard BMT and for the longer and more costly form of BMT with the additional RP sessions showed (a) decreases in health care and legal costs after, as compared to before, treatment; (b) positive cost offsets (or savings); and (c) benefit-cost ratios greater than 1, indicating that health and legal system cost savings (*i.e.*, benefits) exceeded the costs of delivering the BMT treatments. In fact, cost savings from reduced utilization were more than 5 times greater than the cost of delivering the standard 5- to 6-month BMT program. Although adding RP to BMT led to less drinking and better marital adjustment, it did not lead to greater cost savings in health and legal service utilization. The benefit-cost ratio for BMT was 5.97 compared to 1.89 for BMT with RP.

Two cost-benefit analyses [21,22] involving brief interventions by physicians through review of the prevalence of problem drinking, patient specific alcohol effects, worksheet on drinking cues, drinking agreement in the form of a prescription and drinking diary cards for 482 men and 292 women (age 18–65) in Wisconsin, USA, exhibited positive net benefits for patients, the healthcare system as well as society. The intervention, conducted by physicians, included two 15-minute face-to-face counselling sessions one month apart and two 5-minute nurse follow-up phone contacts. The average number of drinks and binge drinking episodes declined at the 6-month follow-up point. Although it declined further during 12–48 month follow-up, the major effect occurred within six months of the intervention. The benefit-cost analysis was performed from two different perspectives: (1) the medical care system perspective, which considered costs to the medical care system and benefits from reductions in future emergency room visits and hospitalizations; and (2) the societal perspective, which considered all costs and benefits to the clinic, patient, and society in general. The benefit-cost ratios were 3.2 and 4.3 for the medical system, and the societal benefit-cost ratios were 5.6 and 39.0 after 12 and 48 months of treatment, respectively.

Mundt and colleagues [23] examined older adults (105 men and 53 women, aged 65 and older) who received a brief intervention by a physician through assessment, feedback, contracting and goal setting. Results indicated a 40% decrease in average weekly alcohol consumption compared to 6% in the control group in 3-month follow-up and maintained significantly lower levels of alcohol consumption and heavy episodic drinking throughout a 24-month observation period. Monetary benefits of $5,241 per patient ($3,260 in healthcare and motor vehicle events: $1,613, life-years lost: $368, and other social consequences: $1,981) were observed for the treatment group as compared to the control group.

In a study of injured patients treated in an emergency department or admitted to a hospital in the USA, Gentilello and colleagues [25] analyzed direct injury-related medical costs and cost-benefits due to screening and brief intervention. It was found that if the brief intervention was offered, the expected cost of screening, intervention, and subsequent emergency department visits and hospital admissions over the next three years was $600 per patient. In the scenario where screening and intervention were not offered, the expected cost of subsequent emergency department visits and hospital admissions was $689 per patient over three years, resulting in an estimated cost savings of $89 per injured patient screened, or $330 for each patient offered a brief intervention. The brief intervention resulted in $3.81 in health care costs saved for every $1.00 spent on screening and intervention.

### 3.2. Pharmacotherapy Studies

**Figure 2.**
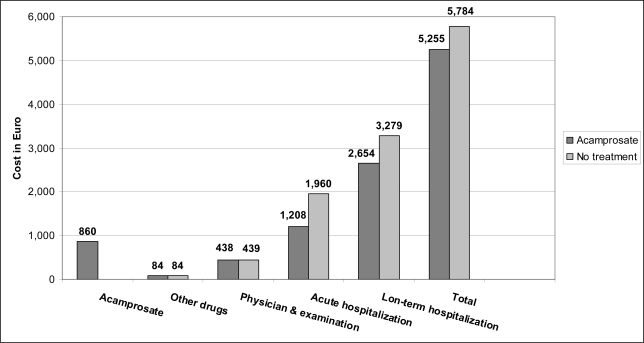
Distribution of costs (in Euros) for acamprosate versus no treatment in the Belgian study [26].

In a study [26] involving 448 alcoholic patients in Belgium, 12-month treatment with acamprosate and 12-month follow-up resulted in net cost savings of 21,301 BEF (528 €) per patient over a 24-month period for acamprosate treatment compared to placebo (Figure 2), due to a fewer acute hospitalizations for detoxification, less liver complications and less institutionalized rehabilitation.

It was estimated that treatment with acamprosate would have an anticipated saving of 70 million BEF (1.74 million Euro) over a two year period, for Belgium. Although this study provided a good estimate of the healthcare costs, there were some limitations. For instance, cost-benefits to the legal system or due to productivity losses were not determined. Also, the cost data were derived from the Belgian health care system, whereas the clinical data come from an Austrian study in which abstinence rates were considerably lower than other European studies [28].

In a study by Portella *et al*. [27] involving the total alcohol-dependent patient population in Spain (estimated to be approximately 627,400 people), it was calculated that treatment of AD (including hospitalization, physician visits, rehabilitation and medication) for the population would cost Spain Pta 42,430 million per year if 50% of AD patients received treatment (Pta 33,944 million if 40% received treatment and Pta 50,917 million if 60% received treatment). The total lifetime benefit for each rehabilitated patient, without any subsequent alcohol-related complications was calculated to be Pta 3,914,680 (23,528 €). The net benefit for the population if 50% of patients were treated under the best-case scenario (29.1% of treated patients become rehabilitated, 25% of rehabilitated patients develop complications) was Pta 303,953 million, and under the worst case scenario (10% of treated patients become rehabilitated, 75% of rehabilitated patients develop complications) was Pta 68,484 million. Varying the percentage of the patient population that would be treated between 40 and 60% produced net benefit ranges between Pta 364,743 million for the best case scenario and Pta 54,87 million for the worst case scenario.

This study has some limitations. While estimating the total lifetime benefit for each rehabilitated patient, it was assumed that there would be no subsequent alcohol-related complications. Again, most of the benefit was attributable to avoidance of indirect costs and non-specific direct costs (Pta 3,409,349), whereas direct health-related benefits were substantially less (Pta 05,331).

## 4. Conclusions

Treatment of alcoholism is associated with a decrease in total health care utilization [29,30] and thus, produces marked economic benefits for most of the treatment options. A present analysis of studies, concerning cost-benefit attributable to AD treatment, revealed that most of the treatment options produce marked economic benefits with the benefit-cost ratio ranging from 1.89 to 39.00. The highest benefit-cost ratios were observed in brief intervention studies [21,22].

All studies involving psychotherapy treatments have reported that major cost-benefits have been achieved in the first six months. The benefits to the healthcare system as well as the society as a whole, of course, continue to increase with time. While in most of the studies, individual counselling and behavioural therapy are quite effective and accrue significant economic benefits, some treatment procedures like interactional couples therapy have resulted in negative benefit-cost ratios due to the high cost of treatment. In addition, it has to be noted that the participants in brief intervention studies included in this analysis [21–24] might not all be alcohol dependent but rather exhibit alcohol abuse.

Although there are relatively more cost-benefit studies on psychotherapy treatments of AD, only two studies were identified that had included a CBA of pharmacotherapy for the treatment of AD. These two cost-benefit studies on pharmacotherapy of AD were related to acamprosate; there were no cost benefit study involving oral or injectable naltrexone, disulfiram or any other drug. Nonetheless, it is important to note from these few available studies that pharmacotherapy treatment of AD accrues economic benefits to the healthcare system as well as to society.

It is important to note that the considerations for benefit-calculations have been different in the reviewed studies. While in some cases, the societal benefits included benefits to the health care system and the legal system; some other studies included savings in other indirect societal costs such as productivity losses.

Reduction of binge drinking/heavy drinking occasions (both regular and irregular) and its associated problems is of overriding importance. A dichotomous criterion of abstinence does not differentiate sufficiently, and is unrealistic, as many have a drink/relapse at one time or another. The reduction of heavy drinking occasions is a key for (a) health outcomes, and also for (b) criminality outcomes. The highest benefit in classical cost-benefit studies will, of course, be achieved if both dimensions are combined, and disability is included in the health outcomes.

Studies on the economic aspects of AD treatment and psychiatric co-morbidity are scarce, although there are a number of studies that demonstrate the efficacy of pharmacotherapy of individuals with AD and co-morbid psychiatric illness [31]. However, the main question here is causality. The main problem is to identify the portion of co-morbidity, which is due to AD. Co-morbidity may be caused by AD, by the other condition, or by a third variable influencing both. We seem to have no way to disentangle these mechanisms. One way to deal with this would be to look into health service utilization, especially in mental institutions, before and after pharmacotherapy of alcohol dependence - e.g., a design where service utilization is measured in the two years before and the two years after treatment and the costs are compared.

The vast majority of identified economic analyses were cost analyses, often mislabeled as “cost-benefit analyses”. The reviewed studies usually compared the costs associated with treatment with the accompanying (economic) savings and estimated net costs (or savings). However, these studies commonly fail to estimate the indirect economic benefit of health effects (*i.e.*, decreases in mortality and improvements in quality of life).

Any new study on AD psychotherapy or pharmacotherapy should include a cost-benefit component. Cost-benefits should be evaluated with the inclusion of all major cost components. Other types of economic components that need to be included are cost-effectiveness (CE) and cost-utility (CU). In CE analysis, the incremental cost of a program from a particular viewpoint is related to the incremental health effects of the program measured in “natural units” such as a symptom score or symptom-free days. The results are expressed as cost per unit of effect in these units. CE components should be measured with the key outcomes (*i.e.*, amount of heavy drinking occasions). In CU analysis, the incremental cost of a program from a particular viewpoint is compared to the incremental health improvement attributable to that program, where the health improvement is measured in quality-adjusted life-years (QALY) gained. Both CE and CU should cover a longer time period (5–10 years), compared to the usual analyses of 6 to 12 months. A new study should thus include a short-term and a long-term component.

Future economic evaluation studies should compare psychotherapy, pharmacotherapy or adjuvant treatments involving combined medications. There is also a need for an intensive study of the interactive effects of a number of combinations of medication other than naltrexone and acamprosate and psychosocial treatment [32].

Any new well-conducted economic study could help define the field further with respect to what should be the standard psychosocial treatment in addition to pharmacotherapy. Adjunctive psychosocial treatment with close follow-up is advisable for AD pharmacotherapy treatment [33]. The strengths of these psychosocial treatment alternatives to enhance medication adherence, preventing attrition, addressing co-morbid problems, fostering abstinence, and targeting the weaknesses of the pharmacologic agent, as well as the characteristics of the target population need to be considered while choosing the type of psychosocial treatment [34].

Future studies also need to consider potential “side effects of treatment” (*i.e.*, subjects may decrease their consumption of alcohol with a treatment, but increase their use of other substances). There is a need to examine the economic effects of AD treatment on other psychiatric disorders, especially if there are compounds that may have positive effects on both AD and psychiatric co-morbid disorders.

## Figures and Tables

**Table 1.** Summary of cost-benefit studies on alcohol dependence treatment.

**Reference**	**Country**	**Population & Sample Size**	**Type of Intervention**	**Type of Cost**
**PSYCHOTHERAPY**				

[19]	USA	36 newly abstinent married male alcoholics		Average costs:
			1. IC	IC $450;
			2. IC + BMT	IC + BMT $857
			3. IC + ICT & 24 month follow-up	IC + ICT $895

[20]	USA	59 couples with a newly abstinent alcoholic husband		Average costs:
			1. BMT	BMT $864,
			2. BMT + RP	BMT + RP $1,640

[21]	USA	482 M & 292 W, age 18–65	BI by physicians & 12 month follow-up; Review of the prevalence of problem drinking, PT specific alcohol effects, worksheet on drinking cues, drinking agreement as a prescription & drinking diary cards	$205 per PT (clinic cost $165.65, PT cost $38.97)

[22]	USA	482 M & 292 W, age 18–65	BI by physicians & 48 month follow-up; Review of normative drinking, PT specific alcohol effects, worksheet on drinking cues, drinking diary cards, drinking agreement as a prescription	$205 per PT (clinic cost $166, PT cost $39)

[23]	USA	105 M & 53 W, age 65+	Brief intervention by physicians & 24-month follow-up assessment, feedback, contracting & goal-setting	$236 per PT (clinic cost $197, PT cost $39)

[24]	USA	Primary care clinics	BI by physicians: 12- & 48-month follow-up	$205 per PT (Screening & assessment $88, training cost $23, intervention cost $55, PT cost $39)

[25]	USA	Injured PT treated in an emergency department or admitted to a hospital (6% of 20,507,601 adult PTs treated for injuries)	Screening + BI	Direct injury-related medical costs (screening + BI) $600 per PT

**PHARMACOTHERAPY**				

[26]	Belgium	448 alcoholic PT	12-month treatment with acamprosate & 12-month follow-up	Per-PT cost: No treatment 5,783 €; Acamprosate 5,255 €

[27]	Spain	Total alcohol-dependent PT population in Spain (approximately 627,400)	Treatment with acamprosate for 1 year, time horizon for benefit was 11–16 years	Spain Pta 42,430 million per year if 50% of affected PT received treatment (Pta 33,944 million if 40% received treatment & Pta 50,917 million if 60% received treatment)

BI: Brief Intervention; BMT: Behavioural Marital Therapy; IC: Individual Counselling; ICT: Interactional Couples Therapy: PT: Patient(s); RP: Relapse Prevention; M: Men; W: Women.

**Table 2.** Net benefit or cost-benefit ratio for alcohol dependence treatment.

**Reference**	**Country**	**Net Benefits**	**Benefit-cost Ratio**
**PSYCHOTHERAPY**			
[19]	USA	Average benefits: IC, $7,581; IC + BMT, $6,681; IC + ICT, (−)$2,248	IC, 20.77 IC + BMT, 8.64 IC + ICT, (−)$2.82
[20]	USA	Average benefits: BMT, $5,053; BMT + RP, $3,365	BMT, 5.97, BMT + RP, 1.89
[21]	USA	$1,151 per PT Savings: Medical $523, Legal cost & MVE $629	3.2 Medical, 5.6 Societal
[22]	USA	$7,985 per PT Savings: Medical $712, Legal cost $102, MVE $7,171	4.3 Medical, 39.0 Societal
[23]	USA	$5,241 per PT Savings: Medical $3,260, MVE $1,613, Life-years lost $368, Other social consequences $1,981	NA
[24]	USA	$7,985 per PT Savings: Medical $712, Legal cost $102, MVE $7,171	4.3 Medical, 39.0 Societal
[25]	USA	$89 for each PT screened; $330 for each PT offered BI	3.81 Medical
**PHARMACOTHERAPY**			
[26]	Belgium	Per PT acamprosate-attributable net cost-savings: € 528 over 2 years	NA
[27]	Spain	Lifetime benefit for each PT, Pta 3,914,680; avoidance of indirect costs & nonspecific direct costs, Pta 3,409,349; avoidance of direct health-related benefits, Pta 505,331	NA

BI: Brief Intervention; BMT: Behavioural Marital Therapy; IC: Individual Counselling; ICT: Interactional Couples Therapy; MVE: Motor Vehicle Event; NA: Not Available; PT: Patient(s); RP: Relapse Prevention.
